# Ventricular Unloading Using the Impella^TM^ Device in Cardiogenic Shock

**DOI:** 10.3389/fcvm.2022.856870

**Published:** 2022-03-23

**Authors:** Adrian Attinger-Toller, Matthias Bossard, Giacomo Maria Cioffi, Gregorio Tersalvi, Mehdi Madanchi, Andreas Bloch, Richard Kobza, Florim Cuculi

**Affiliations:** ^1^Cardiology Division, Heart Center, Luzerner Kantonsspital, Lucerne, Switzerland; ^2^Department of Intensive Care Medicine, Luzerner Kantonsspital, Lucerne, Switzerland

**Keywords:** cardiogenic shock, ventricular unloading, mechanical circulatory support device, Impella, hemodynamics, expert group, review

## Abstract

Cardiogenic shock (CS) remains a leading cause of hospital death. However, the use of mechanical circulatory support has fundamentally changed CS management over the last decade and is rapidly increasing. In contrast to extracorporeal membrane oxygenation as well as counterpulsation with an intraaortic balloon pump, ventricular unloading by the Impella™ device actively reduces ventricular volume as well as pressure and augments systemic blood flow at the same time. By improving myocardial oxygen supply and enhancing systemic circulation, the Impella device potentially protects myocardium, facilitates ventricular recovery and may interrupt the shock spiral. So far, the evidence supporting the use of Impella™ in CS patients derives mostly from observational studies, and there is a need for adequate randomized trials. However, the Impella™ device appears a promising technology for management of CS patients. But a profound understanding of the device, its physiologic impact and clinical application are all important when evaluating CS patients for percutaneous circulatory support. This review provides a comprehensive overview of the percutaneous assist device Impella™. Moreover, it highlights in depth the rationale for ventricular unloading in CS and describes practical aspects to optimize care for patients requiring hemodynamic support.

## Graphical Abstract

Central illustration. Design principles and hemodynamic effects of the Impella™ device. AOP, Aortic pressue; EDP, Enddiastolic pressure; EDV, Enddiastolic volume. 
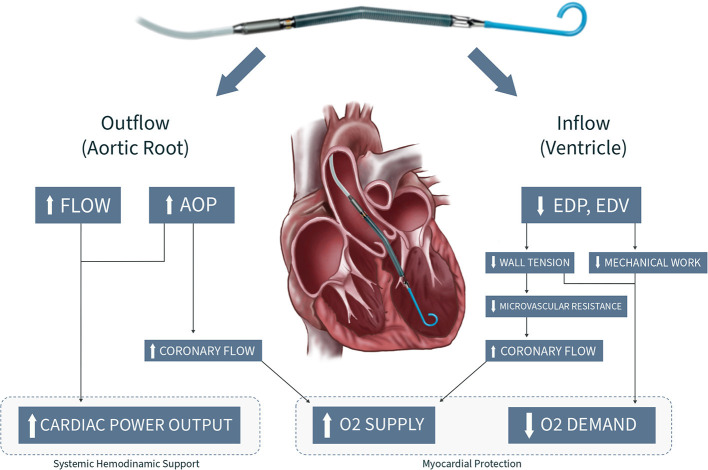

## Introduction

Ventricular dysfunction despite normal or elevated filling pressures associated with hypoperfusion of end organs and tissue hypoxia defines cardiogenic shock (1–3). Acute myocardial infarction (AMI) represents the most common trigger of CS. Other common causes include acute valvular heart disease, ventricular arrythmias, fulminant myocarditis, post-cardiotomy shock and acute or chronic heart failure (HF).

Despite multiple advances including early revascularisation strategies, mortality rates in CS patients remain high (up to 50%) (4, 5). The cornerstones of contemporary CS management include prompt diagnostic workup and initiation of directed therapy aiming to re-establish tissue perfusion and halt the shock spiral. Therapeutic options remained limited for decades, and generally only involved inotropes, vasopressors, ventilatory support and reperfusion therapies. However, the introduction of mechanical circulatory support (MCS) has fundamentally changed CS management over the last decade. This is also reflected by the current European Society of Cardiology (ESC) guidelines with a IIa recommendation for short-term MCS (6). Whereas, particularly in the early MCS era, counterpulsation with an intraaortic balloon pump (IABP) as well as extracorporeal membrane oxygenation (ECMO) represented the preferred devices for refractory CS, the micro-axial Impella™ (Abiomed, Danvers, Massachusetts) is an emerging percutaneous ventricular assist device (pVAD), that has increasingly been used in Western countries (7). In fact, there is a paradigm shift in CS management, which not solely aims for enhancing coronary blood flow (IABP) and maintaining systemic perfusion (ECMO), but also incorporates ventricular unloading ultimately aiming for myocardial recovery.

With this background, this comprehensive review highlights the rationale for ventricular unloading in CS. Moreover, it summarizes important practical aspects, possible complications and current evidence one needs to be aware of, when managing patients requiring hemodynamic support with an Impella™ device.

## Pathophysiology of Cardiogenic Shock

CS represents a complex interplay between the heart and all other organ systems. Rapidly deteriorating myocardial contractility results in a spiraling process of ventricular dysfunction, hypotension, reduced venous return and diminished coronary perfusion leading to pulmonary congestion, hypoxia, decreased organ perfusion and worsening ischemia (3). Compensatory peripheral vasoconstriction initially improves coronary and peripheral perfusion. However, it contributes to increased cardiac afterload that overburdens damaged myocardium further diminishing circulating oxygenated blood flow (3, 8). Systemic hypoperfusion triggers endothelial dysfunction, systemic inflammatory response syndrome (SIRS) and coagulopathies, which all promote multiorgan dysfunction syndrome (MODS) (3). Activated systemic inflammatory mediators (e.g., interleukins, TNF-alpha) result in vasodilation and additional hypotension. Consequently, these mechanisms add up to the high mortality associated with cardiogenic shock (9).

## Role of pVADs in Cardiogenic Shock

The management of CS should focus on preventing and reversing organ failure through hemodynamic resuscitation and simultaneously addressing treatable causes.

Vasoactive and inotropic drugs, especially those with adrenergic mechanisms, have the burden to increase afterload, aggravate myocardial ischemia and trigger arrhythmias, which all ultimately worsen the patient's prognosis. Therefore, they must be cautiously titrated in the setting of CS (10, 11). Consequently, in patients presenting with impeding or already established cardiogenic shock, immediate MCS may be the first choice to rapidly re-establish stable hemodynamics and potentially prevent related MODS.

To date, three basic concepts have commonly been used for percutaneous MCS in acute CS management: (1) counterpulsation using the IABP, (2) ventricular unloading provided by Impella™ technology or by the pulsatile PulseCath iVAC2L device, and (3) veno-arterial extracorporeal membrane oxygenation (VA-ECMO) circulatory support. The mechanisms and hemodynamic effects of currently available MCS are highlighted in Table 1.

**Table 1:** Characteristic features of cardiogenic shock.

**Clinical features of cardiogenic shock**
Myocardial contractile dysfunction
• Low CO (CI <2.2L/min/m^2^) despite normal or elevated pre-load (LVEDP ≤ 15mmHg)
Prolonged hypotension requiring support by catecholamine
• SBP <90mmHg for ≥ 30 minutes
Clinical signs of impaired end-organ perfusion^*^
• Cool extremities • Altered mental status • Oliguria (<30 ml/h) • Rising lactate levels (>2.0 mmol/L)
Pulmonary congestion

* *Despite normovolemia or hypervolemia*.

*CO, Cardiac output; CI, Cardiac index; LVEDP, Left ventricular enddiastolic pressure; SBP, Systolic blood pressure*.

## The Impella™ Device

The Impella™ is a percutaneous, microaxial pump that continuously draws blood from its inlet inside the ventricle and expels it in the ascending aorta (*Central Illustration*) (12–15). Owing its properties, the Impella™ unloads the left ventricle (LV) while simultaneously augmenting cardiac output (CO). The power connections for the pump motor and sensors are contained inside the 9F guiding catheter. The end of the catheter is connected to an external console consisting of an integrated controller for the pump and purge system. Unlike IABP, the Impella™ does not require ECG or arterial waveform triggering, facilitating stability even in the setting of ongoing tachyarrhythmias or electromechanical disassociation.

Currently, four devices are available: Impella™ 2.5, Impella™ CP and Impella™ 5.0/5.5 and Impella™ RP (Table 2). While the Impella™ 2.5 and CP are inserted percutaneously, the Impella™ 5.0 requires a surgical cutdown for insertion. Thus, in many institutions, the Impella™ 2.5 or CP reflect the first choice for mechanical support. The Impella™ RP is a 22 French, three-dimensional catheter-based micro-axial pump approved for use in acute right heart failure (RHF). The inflow of the Impella™ RP is positioned in the inferior vena cava (IVC) and the outflow in the pulmonary artery (PA) expelling blood from the IVC into the PA at a rate of up to 4.6 L/min.

**Table 2:** Impella devices and pump characteristics.

	**IMPELLA 2.5**	**IMPELLA CP**	**IMPELLA 5**	**IMPELLA RP**
Access	Percutaneous	Percutaneous	Surgical	Percutaneous
Access site	Femoral; (axillary)	Femoral; (axillary)	Axillary; Femoral/ascending aorta	femoral vein
Guiding catheter size	9 F	9 F	9 F	11 F
Motor size	12 F	14 F	21 F	22 F
Introducer size RPM	13 F peel away	14 F peel away	23 F peel away^*^	23 F peel away
RPM (max.)	51,000	46,000	33,000	33,000
Duration of support (days)^#^	5	5	10	14

* *Surgical cutdown and insertion through a Dacron graft (8-10 mm) recommended*.

# *European approval (CE Mark)*.

*F, French; RPM, Revolutions per minute*.

## Hemodynamic Effects of pVADs and the Concept of Ventricular Unloading

### From Ventricular Venting to Unloading

Ventricular “venting” has been used in cardiac surgery for decades and refers to strategies to treat ventricular distension and prevent pulmonary edema occurring during cardiopulmonary bypass support and VA-ECMO (16). Different techniques have been applied including trans-septal septostomy (17), and surgical placement of an LV vent. Counterpulsation using is an alternative percutaneous option, thought to decompress the LV.

Since ventricular volume and pressure overload represents the hallmark of patients in CS, the concept of ventricular “venting” was adopted for CS patients. For many years, the IABP was the preferred and only support device for patients presenting with AMI and CS. However, efficacy of circulatory support by IABP is often insufficient considering the results of the randomized IABP-SHOCK II trial and a large meta-analysis with 2,123 patients showing no mortality reduction (18, 19).

In contrast to ventricular “venting”, “unloading” is an active process reducing volume and pressure by pumping blood from the right or left ventricle to the pulmonary artery or aortic root, respectively. Historically, ventricular unloading in CS has been technically challenging, and a series of devices including the TandemHeart remained prototypes or never found widespread clinical use due to their complicated mode of implantation. The introduction of the catheter-based ventricular assist device Impella™ helped to overcome some of those hurdles.

### Hemodynamic Effects of the Impella Device

**Figure 1:**
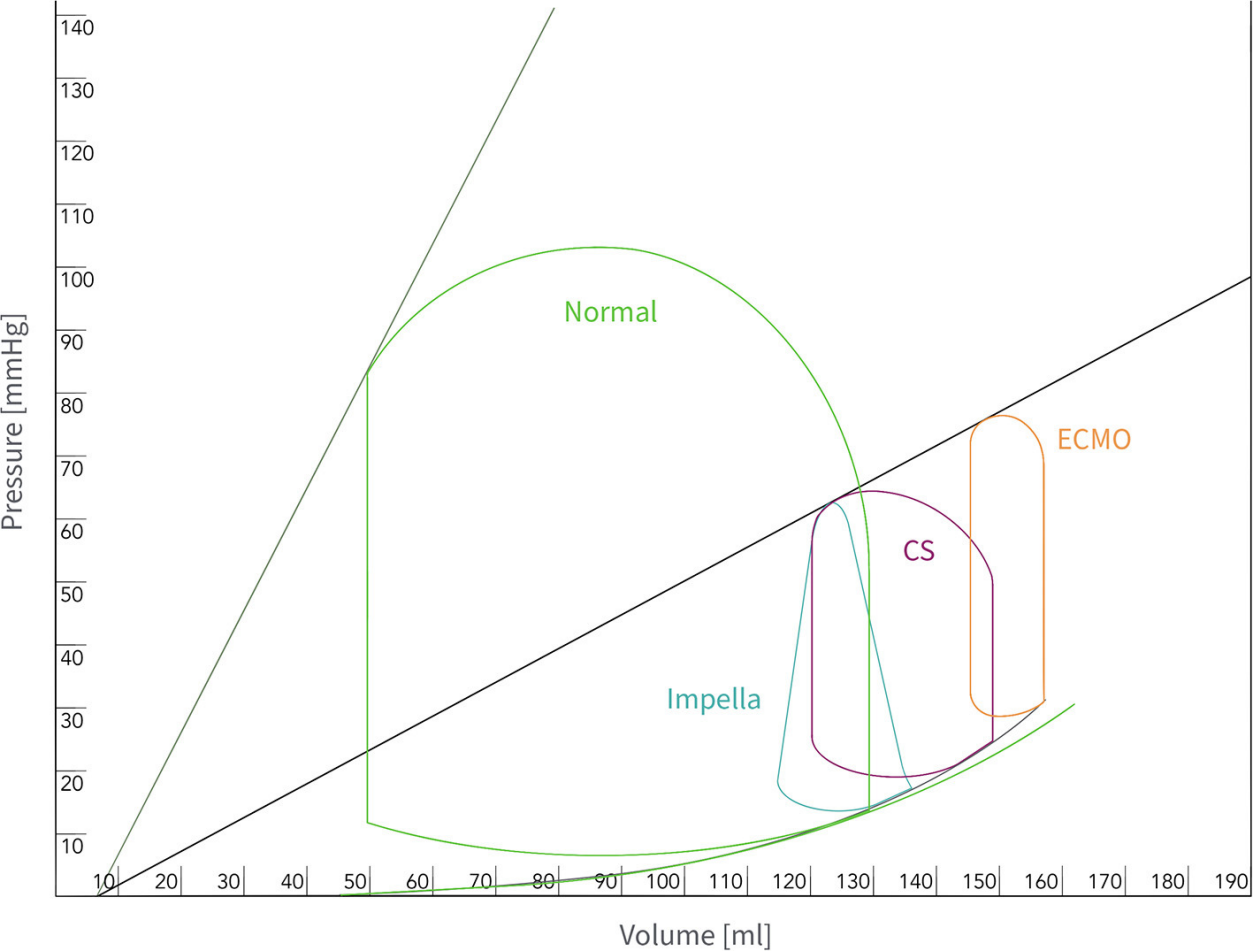
Pressure-volume relationship: Normal conditions (CO 5l; green), CS (CO 3l, PAWP 27 mmHg; purple), CS on VA-ECMO support (3l flow, orange); CS on Impella™ CP support and “P” Level 9 (4 l flow; turquoise). The pressure-volume area represents an estimate of mechanical work performed by the ventricle. The pressure-volume area is only reduced with the Impella™, thus decreasing LV work. *CO, Cardiac output; CS, Cardiogenic shock; LV, Left ventricular; PAWP, Pulmonary artery wedge pressure; VA-ECMO, veno-arterial extracorporeal membrane oxygenation*.

There are four physiologic effects of left sided Impella support: (1) With the inflow of the device drawing blood directly from the ventricle (ventricular unloading), it reduces ventricular end-diastolic volume (EDV) and pressure (EDP) (20). Decreasing EDV and EDP leads to a reduction of myocardial wall tension and workload, both of which diminish myocardial oxygen demand (21–24). This is further highlighted by the progressive loss of isovolumic phases during increasing Impella support illustrated by the conversion of pressure-volume loop into a triangular shape (Figure 1). (2) The outflow of the Impella™ device in the aortic root provides active flow increasing mean arterial pressure (AOP), diastolic pressure, CO and thus cardiac power output (20, 25, 26). If properly placed, the outflow of the device resides just above the aortic valve plane and provides before mentioned systemic pressure augmentation in correlation to the level of Impella support (“P” level) (27). (3) The synergistic effect of increased mean AOP and decreased myocardial wall tension leads to augmented coronary flow, thus improving myocardial oxygen supply. Overall, the Impella device favorably alters the balance of myocardial oxygen demand and supply and therefore improves the heart's ability to survive ischemic challenges (28, 29).

In contrast, VA-ECMO decreases preload, but at the same time substantially increases afterload, which adversely impacts myocardial oxygen consumption. While a healthy LV can cope with increased afterload by recruiting more contractility, the impaired LV in CS may further decompensate leading to a vicious cycle of mechanically driven injury with worsen pulmonary congestion, acute lung injury and pulmonary hemorrhage, thereby worsening cardio-pulmonary function (30, 31). (4) Left ventricular Impella™ support results in decreased pulmonary capillary wedge pressure (PCWP) and a secondary reduction in RV afterload (14). (Table 3, Central Illustration).

**Table 3:** Technical properties of percutaneous circulatory assist devices.

	**IAPB**	**IMPELLA 2.5**	**IMPELLA CP**	**IMPELLA 5.0**	**VA-ECMO**
Mechanism	Aorta	LV → aorta	LV → aorta	LV → aorta	RA → aorta
Cannula size (Fr)	7–8	13–14	13–14	22	14–16 arterial 18–21 venous
Flow (L/min)	0.3–0.5	1.0–2.5	3.7–4.0	5.0	3.0–7.0
Pump mechanism	Pneumatic	Axial flow	Axial flow	Axial flow	Centrifugal
Stable rhythm	Yes	No	No	No	No
Implantation time	+	++	++	++++	++
Risk of ischemia	+	++	++	++	+++
Anticoagulation	+	+	+	+	+++
Cardiac power	↑	↑↑	↑↑	↑↑	↑*↑↑*
Afterload	↓	↓	↓	↓	↑*↑↑*
MAP	↑	↑↑	↑↑	↑↑	↑↑
LVEDP	↓	↓↓	↓↓	↓↓	↔
PCWP	↓	↓↓	↓↓	↓↓	↔
LV preload	–	↓↓	↓↓	↓↓	↓
Coronary perfusion	↑	↑	↑	↑	–

*IABP, intraaortic balloon pump; VA-ECMO, veno-arterial extracorporeal membrane oxygenation; LV, Left ventricle, RA, Right atrium; MAP, Mean arterial pressure; LVEDP, Left ventricular end-diastolic pressure; PCWP, Pulmonary capillary wedge pressure*.

### Systemic Hemodynamic Support

**Figure 2:**
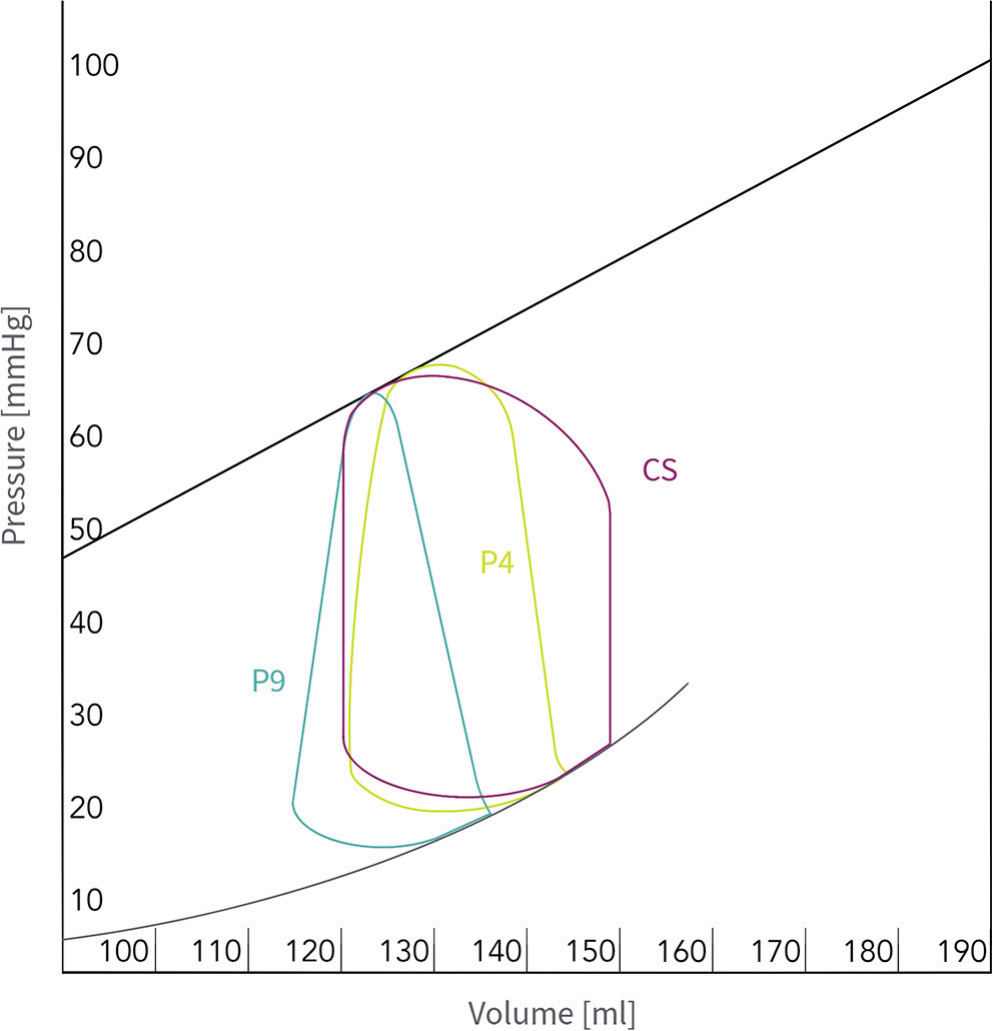
Pressure-volume relationship on Impella™ CP support and different performance (“P”) level settings: The evolution of the pressure-volume relationship before (CS: CO 3l, PAWP 27 mmHg; purple) and after support with “P” Level 4 (2.5 l flow; green) and “P” Level 9 (4 l flow; turquoise). *CO, Cardiac output; CS, Cardiogenic shock; PAWP, Pulmonary artery wedge pressure*.

The Impella augments both flow and pressure in the aorta leading to improved cardiac power output and increased AOP. The actively generated forward flow depends on (1) the specific device (Table 3), (2) the performance (“P”) level setting and (3) the pressure gradient across the aortic valve. Higher “P” level settings or lower pressure gradients result in higher flow augmentation (20, 25, 26, 29, 32). Importantly, the increase in systemic CO results from the net effect of native CO reduction after ventricular unloading and the forward flow contribution of the Impella™ pump. As a consequence, the mean AOP correlates with the Impella™ support and can be modified by changes in the “P” level setting, as highlighted in Figure 2.

### Myocardial Protection: Augmenting Coronary Flow and Increasing O_2_ Supply

Coronary artery flow is proportional to the ratio of AOP and microvascular resistance. By drawing blood directly from the ventricle, the Impella™ reduces maximum wall tension and microvascular resistance. Therefore, the synergistic effect of increased AOP and the reduction of microvascular resistance with increasing Impella support levels lead to a subsequent augmentation of the coronary flow (15, 28). Of note, the constant flow of the Impella device provides more sustained augmentation throughout the diastolic period. In contrast, the IABP deflates in late diastole, which leads to transient pressure increase only early in diastole but this augmentation reverses just before systole, lowering end-diastolic pressure. For instance, the positive effects of the Impella™ on coronary microcirculation has been illustrated in a case report from Agel et al. (33). On nuclear perfusion imaging, they demonstrated adequate myocardial perfusion through collaterals while on Impella™ support in a patient with severe three vessel disease, including complete occlusion of the right and left circumflex coronary artery (33).

### Ventricular Unloading: Decreasing O_2_ Demand

Myocardial oxygen demand is determined by the amount of mechanical work the muscle produces and the amount of myocardial potential energy, which is related to wall tension (21–24, 28). By drawing blood from the ventricle, the Impella™ reduces total filling volume and pressure, which leads to a reduction in stroke volume according to the Frank-Starling mechanism: “If the heart fills less, it expands less and reduces its subsequent stroke output, which corresponds to a reduction in mechanical work” (Central Illustration).

### Oxygen Demand-Supply Ratio

The reduction in EDP, EDV and wall stress lead to reduced microvascular resistance and increased myocardial perfusion (increasing myocardial oxygen supply). In addition to this perfusion effect, ventricular unloading results in reduced mechanical work and potential energy (reduced myocardial oxygen demand). This impact is expressed in the pressure-volume (PV) loop by a leftward shift in its position and an overall reduction in its area (Figure 1). Of note, while significant reduction of ventricular work as well as end-diastolic pressure and volume was shown with the Impella, changes in the same parameters with the IABP were not significant (28).

## Acute Right Heart Failure and Right Ventricular Unloading

RHF is a characterized by the inability of the right ventricle (RV) to sustain pulmonary flow caused by increased RV afterload (e.g., acute pulmonary embolus, severe hypoxia, acidemia, or increased intrathoracic pressures) or decreased RV contractility (e.g., RV ischemia, myocarditis, post-cardiotomy CS, or LVAD support) (34–36). RHF is associated with high morbidity, mortality, and longer hospital length of stay (37). The thin walled RV differs markedly from the LV in architecture, mechanics, metabolism, and recovery from injury (38). The RV is exquisitely susceptible to failure under conditions of ischemia and pressure overload. However, the RV is remarkably resilient and tends to recover once hemodynamics improve and the underlying insulting cause is eliminated. But in some patients RHF persists and, similar to LV related shock, outcome in patients requiring multiple and prolonged inotropic and vasopressor support is poor (10, 11). Moreover, 10–40% of patients undergoing isolated left ventricular assist device (LVAD) implantation experience some degree of RHF (39). While RHF associated with LVAD insertion may be partially caused by the underlying cardiomyopathy, the pathophysiology of RHF after LVAD implantation is complex.

In this context, the Impella™ RP provides an opportunity for mechanical support in the downward spiral of refractory RHF and may serve as a bridge to recovery or heart transplant. Since survival after impella™ RP insertion strongly depends on timing and patient selection (37, 40, 41), early identification of RHF and careful consideration of patient's clinical status and comorbidities is key to obtain the best clinical outcomes. Device implantation requires some expertise and, in contrast to LV pumps, can only be performed under fluoroscopy guidance. Frequent monitoring of RV function using echocardiography and pulmonary artery catheter measurements is crucial to guide Impella™ RP therapy. Based on the inclusion and exclusion criteria of the RECOVER RIGHT trial (37) and a series of smaller clinical studies and case series (42–45), a dedicated checklist for patient selection has been proposed, see Table 4.

**Table 4:** Impella RP heart pump patient selection recommendations.

**Clinical conditions in which the Impella RP is not recommended**
Active infection with positive blood cultures
RA, RV or PA thrombus
Mechanical valves in the right heart^*^
Unrepaired ASD, PFO, or aortic dissection
PA conduit
Anatomic abnormalities precluding insertion
Moderate to severe pulmonary valve stenosis or insufficiency
Severe pulmonary hypertension (PAPs > 60mmHg)
Documented DVT and/or presence of IVC filter
Patients on right-sided support or ECMO
Allergy or intolerance to contrast
HIT or sickle cell disease
**Definition of RHF**
CI <2.2 l/min/m^2^ despite continuous infusion of high dose inotropes^#^ and any of the following: • CVP > 15 mmHg or • CVP/PCWP > 0.63 or • Moderate to severe global RV dysfunction on echocardiography defined as one of the following criteria: ° Global RV hypokinesis ° TAPSE score of ≤ 14 mm °RV diameter at basis >42 mm ° RV short axis (or mid-cavity) diameter >35 mm

*Table adapted from Abiomed^®^ recommendations for Impella RP patient selection*.

* *Presence of a tricuspid ring or bio-prosthesis is not a contra-indication, but it may result in a difficult implantation depending on the valve strut orientation within the RVOT*.

# *Dobutamine of ≥ 10 μg/kg/min or equivalent for more than 15 min (120 min for milrinone) and/or administration of more than one inotrope/vasopressor*.

*ASD, Atrial septal defect; CVP, Central venous pressure; PCWP, Pulmonary capillary wedge pressure; DVT, Deep vein thrombosis; HIT, Heparin induced thrombocytopenia; PA, Pulmonary artery; PAPs, Pulmonary artery systolic pressure; PFO, Persistent foramen ovale; RA, Right atrium; RV, Right ventricle*.

## Current Evidence

The only two randomized clinical trials comparing the Impella™ vs. IABP have both been neutral with respect to survival. However, both were underpowered, the ISAR-SHOCK trial mainly targeted hemodynamic improvements (46). The small IMPRESS trial also showed similar outcomes with both Impella CP and IABP in patients with ST-elevation myocardial infarction (STEMI) and CS undergoing primary percutaneous coronary intervention (PCI) (47). One must take in account that this trial included critically ill patients and the major cause of death was anoxic brain injury, suggesting that mechanical hemodynamic support may be of limited utility in this patient cohort. Also, the trial was underpowered (47). Although, some centers have reported better survival rates in CS after implementation of a comprehensive shock protocol using pVADs (48, 49), the use of Impella™ has been associated with higher risks of bleeding, stroke, and death, as well as higher costs compared to IABP in propensity-matched analyzes from registry data (7, 50, 51). However, confounding due to the use of pVADs in sicker patients cannot be ruled out (51). Despite neutral results in randomized clinical trials and the remaining high mortality rates in this severely ill population there is some evidence that the use of larger Impella™ pumps (e.g., Impella™ CP), the initiation of Impella™ prior to PCI and its use in patients without cardiac arrest may be correlated with outcome improvements (52).

In comparison to VA-ECMO, the incidence of major complications, such as bleedings, might be lower with Impella™ use (53). The data supporting the use of RV pVADs, namely the Impella™ RP, is even more limited and randomized data is not yet available. The RECOVER RIGHT study was the first to suggest feasibility and safety of the Impella™ RP in patients with severe RHF (13). A series of recent studies indicated possible clinical benefit with the Impella™ RP demonstrating 30-day survival rates of of 64–72% (37, 40, 41). However, the survival rate was much lower among patients in whom Impella™ RP was implanted as salvage support (41). This caused the U.S. Food and Drug Administration to issue a warning advice. This controversy highlights the need for proper patient selection and early initiation of hemodynamic support.

## Case Selection

### Left Ventricular Impella™ Devices

Contraindications to the placement of the LV Impella™ include mechanical aortic valve, LV thrombus, moderate to severe aortic regurgitation, and severe obstructive peripheral arterial disease.

Visualization of the ventricle before implantation excluding the presence of a thrombus is recommended if time permits using a bed-side echocardiogram. Thrombus may be sucked up by the impeller and interrupts its proper functioning. As with any other catheter placed in the LV, the Impella™ catheter may furthermore dislodge thrombus, potentially causing systemic embolization. Moderate to severe aortic regurgitation (AR) is a relative contraindication. Only a competent aortic valve separating the LV and aorta allows optimal antegrade Impella™ flow. In patients with relevant AR, AOP augmentation by the Impella™ may worsen AR and LV dilation. Given concerns regarding compromise of the remaining valvular orifice and worsening hemodynamics with the introduction of the Impella™ catheter, aortic stenosis (AS) has been considered an exclusion criterion in clinical trials. Also, crossing of a severely stenotic aortic valve with the impella device might be very challenging. Despite these concerns, feasibility of Impella™ insertion in severe AS before high-risk PCI, during balloon valvuloplasty or transcatheter aortic valve replacement (TAVR) and bail-out use as a bridge to TAVR in CS has been demonstrated in several reports (54–60). Peripheral artery disease (PAD) may not be an absolute contraindication for the Impella™ insertion, nevertheless its presence and extent need to be considered prior to device implantation. Femoral angiography in an ipsilateral projection prior to Impella™ insertion to assess puncture height and anatomical suitability of the iliac and femoral arteries allows to identify prohibiting PAD and may prevent access site complications and limb ischemia. Additionally, ultrasound guidance helps to find the ideal puncture site and avoid impeding calcifications. In afflicted patients, alternative access routes (trans-subclavian or -axillary) may be evaluated. However, to avoid complications prudent access site management is crucial. Several strategies for closure of the arteriotomy after removal of the device are utilized dependent on availability and local experience. Manual compression is a cost-effective, although time intensive means to achieve hemostasis. Femoral compression systems (e.g., using FemoStop, Abbott Vascular) can be applied to avoid bleeding after device removal. Latest generation of the Impella™ sheaths allow advancement of a wire for sheath exchange or placement of closure devices, such as the MANTA^®^ 14 F device (Teleflex Inc., Morrisville, North Carolina) or the Perclose ProGlide™ suture-mediated closure System (Abbott Vascular Inc., Santa Clara CA, U.S.A.). In selected cases at high risk for bleeding or ischemic complications surgical removal might be safest.

### Impella™ RP

As for the left ventricular devices, only a competent pulmonary valve allows optimal forward flow. However, a certain degree of pulmonary valve regurgitation is often present in the setting of acute RHF and elevated pulmonary artery pressures. Albeit significant tricuspid valve regurgitation (TR) often accompanies RHF, hemodynamic effects of the Impella™ RP are usually not affected if the pulmonary valve is competent. So far, TR represents a relative contraindication for Impella™ RP implantation according to the manufacturer. However, TR might improve after RV unloading, particularly if TR is secondary to annular dilatation in the setting of acute RHF. Therefore, TR should rather be seen as a warning sign than as an absolute contraindication.

## Indications for Ventricular Unloading

In addition to its application in high-risk PCI and cardiogenic shock complicating AMI, the Impella™ technology has been successfully introduced in a broad variety of clinical scenarios requiring left or right ventricular support. Indication for ventricular unloading and issues to be considered when selecting patients for pVAD support are depicted in Table 5.

**Table 5:** Indication for ventricular unloading.

**Clinical scenarios requiring left or right ventricular support**		
*Emergency interventions*	*Planned interventions*	
AMI complicated by CS	High-risk PCI	
Post-cardiac surgical (bi)ventricular failure	Catheter ablations of VT	
Fulminant myocarditis	High-risk bypass surgery	
Advanced heart failure Valvular heart disease (e.g. AS) with severe LV dysfunction		
Hemodynamic deterioration after TAVR		
**Clinical conditions to be considered in patient selection with CS**		
*Coronary artery disease* *and treatment considerations*	*Clinical considerations*	*Hemodynamic considerations*
Large LAD or RCx related STEMI Adequate peripheral access	Comorbidities (e.g. expected neurological outcome, diabetes, renal failure, PAD)	SBP <90mmHg and/or inotropic pressure-dependance Tachycardia (HR >100/min)
Preferably initiate Impella support before PCI	Bleeding risk (ACT 160–180 s)	LVEDP >30–35 mmHg

*ACT, Activated clotting time; AMI, Acute myocardial infarction; AS, Aortic stenosis; CS, Cardiogenic shock; HR, Heart rate; PCI, Percutaneous coronary intervention; STEMI, ST-elevation myocardial infarction; TAVR, Transcatheter aortic valve replacement; VT, Ventricular tachycardia*.

Timely implantation is often key. Considering the rapidly progressing shock spiral, early identification and treatment are crucial to increase chances of survival. This seems underlined by observational data suggesting that Impella™ implantation before revascularization maximizes the potential benefit (61) and that survival decreases by about 10% for every 60 min of delay (49).

### Mechanical Support in Coronary Bypass Surgery and Post-cardiotomy Cardiogenic Shock

Nowadays most patients presenting with CS secondary to myocardial infarction (MI) are revascularized percutaneously. However, there is a subset of patients who need to be referred for urgent or emergent coronary artery bypass grafting (CABG). In a US registry, 129 (2.3%) patients with MI and CS undergoing CABG had MCS inserted (62). Most of these patients were bridged to surgery with an Impella™ device. Although, operative mortality in this emergency setting was very high (37.2%), the data suggests that there may be some benefit to instituting MCS prior to CABG in this very high-risk group of patients. Also, there are reports of prophylactic pVAD utilization in high-risk patients undergoing off-pump CABG to minimize cardiovascular instability following heart positioning for proper suturing of coronary anastomoses (63–65).

Overall, 0.2–9% of the patients undergoing cardiac surgery experience post-cardiotomy CS, which is associated with a high mortality (66, 67). Early data from 24 patients, who could not be weaned from cardiopulmonary bypass or were hemodynamically unstable and therefore needed support with the Impella™ Recover device (providing 3–4 L/min flow), showed improved outcome compared to IABP alone, if the heart was able to pump >1l/min (68). Thomas et al. (69) reported the first successful use of an Impella™ 5.0 L/min for post-cardiotomy CS after coronary artery bypass grafting and bioprosthetic aortic valve replacement. Support was maintained for 7 days. Noteworthy, no damage to the bioprosthetic aortic valve was seen. Despite these promising reports, VA-ECMO is still much more commonly employed in patients with post-cardiotomy CS_and further studies are necessary to support the use of the Impella™ device in the setting of cardiac surgery.

## Complications Associated With Impella™

Overall, the type of complications related to the use of the Impella™ device are similar to those encountered with the IABP. The most common complications include limb ischemia, vascular injury and bleeding requiring blood transfusion (15). The reported incidence of limb ischemia ranges from 0.07–10% and for significant bleeding from 0.05 to 54% (70). The risk of bleeding is also related to the administration of antithrombotics (e.g., unfractionated heparin), thrombocytopenia or consumption of coagulation factors (e.g., von-Willebrand factor) related to shear-stress with the impeller. Moreover, shear stress from the impeller (especially at very high “P” levels) can lead to clinically relevant hemolysis, which in worst case scenario can cause renal failure. This phenomenon has been observed in 5–10% of patients during the first 24 h on Impella™ support. The risk of hemolysis and aortic valve injury may be diminished by proper positioning of the inlet cannula, and thus limited flow turbulences.

Ischemic or hemorrhagic cerebrovascular accidents following Impella™ insertion have also been reported (2.4–6.3%) (70, 71). As with any percutaneous device, there is a risk of access site infection and sepsis, which increases with the duration of support. In the early experience, device migration and malfunction rarely led to injury of the aortic valve or ventricle causing tamponade due to LV perforation. Also, mitral regurgitation secondary to injury of the papillary muscles or chordae have been reported (32). Finally, the pigtail end of the Impella™ within the LV can provoke ventricular arrhythmias potentially further impairing CO and deteriorating CS.

## Escalation of Support and Combination of Impella™ With Other Devices

MCS is a key element of most modern cardiogenic shock care pathways (3). It is recommended to define triggers for initiation of MCS, choice of MCS modality, and escalation steps in CS patients. Such pathways should certainly be tailored to local MCS availability and experience. Irrespective of MCS modality, the adequacy of hemodynamic support and ventricular unloading needs to be closely monitored.

Adequate monitoring including a pulmonary artery catheter (measurement of central venous pressure (CVP), PCWP, CO) in combination with standard clinical measures such as blood pressure, lactate and urine output is mandatory. Frequent echocardiographic assessments of LV/RV size, function, and aortic valve opening can help to optimize pharmacologic treatment and guide escalation of mechanical circulatory support.

According to the anticipated degree of support required, the Impella™ 2.5, Impella™ CP, or the surgically implanted Impella™ 5.0/5.5 may be considered. The support requirements depend on body size as well as the degree of hemodynamic compromise. It is crucial to be aware, that due to the Anrep effect the intended Impella™ flow cannot be simply added to the pre-insertion native CO. Reduced contractility of the ventricle after pump insertion will result in a smaller total CO than expected. Among patients with profound LV dysfunction an Impella™ pump might unload the LV to the point of continuous aortic valve closure resulting in a non-pulsatile arterial curve on the monitor.

The Impella™ 2.5 and CP, which can rapidly be inserted percutaneously, are usually the first choice in the setting of CS. However, in patients with severe LV failure, low CO might persist for several days, sometimes even weeks or months. In such cases, upgrading to a larger device (e.g., Impella™ 5.0 or 5.5) might be a wise strategy (72). With these devices the patient can even be ambulated while awaiting recovery, cardiac transplant or LVAD implantation.

Simultaneous RHF results in reduced LV preload and therefore limits the flow of the LV Impella by recurrent suction events, which necessitates down-titration of the Impella™ pump power level. In case of inadequate hemodynamic response after LV unloading and recurrent suction alarms irresponsive to volume challenge, the insertion of an VA-ECMO or Impella™ RP may be considered. The latter augments RV output and therefore increases LV preload (BiPella approach), which in turn improves CO. Although, there are only small case series available (45, 73–78), the BiPella approach seems to be feasible and safe und might be used as a salvage treatment modality for refractory biventricular failure. VA-ECMO might be primarily considered in the setting of inappropriate oxygenation due to acute lung congestion or MODS. Also, VA-ECMO implantation might be evaluated in case of refractory shock and inadequate support from the Impella™.

Conversely, some patients on VA-ECMO support may benefit from additional LV unloading by an Impella™ device. Although potentially lifesaving in patients with CS, VA-ECMO burdens the already impaired LV by increasing afterload. This may further compromise the LV contractile function due to ventricular distension and impaired myocardial blood flow (79, 80). When deployed in combination with VA-ECMO, the Impella™ (ECMELLA approach) reduces filling pressures, ventricular distension, and maintains flow from the LV to the aorta even in the absence of LV ejection and a closed aortic valve (81–83). There is data proposing the combined use of VA-ECMO with an Impella™ device in severe CS cases to unload the LV, facilitate myocardial recovery and improve clinical outcomes. Yet, the evidence supporting the ECMELLA approach derives only from observational studies and has accordingly some limitation (81–84). Also, the increased risks of hemorrhagic and vascular complications due to the additional large bore vascular access required need to be considered.

Finally, there are small case series endorsing the combination of an Impella™ device and IABP as a bail out strategy in refractory CS (28, 85). However, further clinical investigations will be needed to assess if the combination of LV unloading and counterpulsation using the IABP brings any incremental physiological and clinical benefit. Possible combinations of different MCS devices, their indications, clinical effect and possible pitfalls are depicted in Table 6.

**Table 6:** Combination of Impella™ with other devices.

	**Indication**	**Effect**	**Limitations**
Impella™ + VA-ECMO (ECMELLA)	- Gas exchange failure - Refractory CS/inadequate support - Concomitant RHF^*^ following LV Impella™ insertion - RHF and severely elevated PVR - Recurrent tachyarrhythmias - Pulmonary hemorrhage	Hemodynamic support ↑ Oxygenation ↑ and CO_2_ elimination RV unloading	- Access site complications - Increased LV afterload - Bleeding diathesis - Post-implantation management complexity - Cost-intensive
VA-ECMO + Impella™ (ECMELLA)	- LV stasis with thrombus formation - Pulmonary failure due to high PAP - LV distension - Myocardial ischemia	LV/RV unloading Myocardial perfusion ↑	- Reduction of VA-ECMO flow required - Post-implantation management complexity - Cost-intensive
Impella™ + Impella™ RP (BiPella)	- Biventricular failure - Concomitant RHF^*^following LV Impella™ insertion	RV output ↑ LV suction alarms ↓ (at maximal LV pump speed) CO ↑	- Implantation of Impella™ RP requires expertise and fluoroscopy guidance - Limited efficacy in severely elevated PVR - Cost-intensive
Impella™ + IABP	Refractory CS	Myocardial perfusion ↑ Oxygen demand–supply ratio ↓	- Limited hemodynamic support - Possible overall reduction in the Impella flow

* *Which is not volume responsive*.

*CS, Cardiogenic shock; CO, Cardiac output; IABP, intraaortic balloon pump; VA-ECMO, Veno-arterial extracorporeal membrane oxygenation; LV, Left ventricle, RV, Right ventricle; PAP, Pulmonary artery pressure; PVR, Pulmonary vascular resistance*.

## Future Perspectives

With respect to the limited evidence supporting the use of the Impella™, especially in patients with AMI and/or CS, there are several trials in progress. For instance, the DanGer Shock trial (ClinicalTrials.gov Identifier: NCT01633502) is a prospective, multicenter, open-label trial randomizing AMI patients with CS 1:1 to Impella™ CP support or current guideline-driven therapy with a planned enrollment of 360 patients (86).

Also, following encouraging pre-clinical studies (87, 88), which suggest a reduction in infarct size by applying early ventricular unloading in patients with AMI, the STEMI-DTU trial (ClinicalTrials.gov Identifier: NCT03947619) will study the impact of ventricular unloading by the Impella™ device during 30 min before primary PCI on infarct size in patients with acute anterior MI.

Besides new treatment concepts, also new devices are currently under investigation or development. Since the actual versions of percutaneously implanted LV Impella™ devices bear the risks of bleeding and vascular injury, there have been efforts to downsize the catheter size. There is now a nine french device – the Impella™ ECP – under clinical investigation (ClinicalTrials.gov Identifier: NCT04477603). Also, new devices allowing LV unloading with integrated batteries are under development, ensuring long-term hemodynamic support for several months and enabling patients to leave the hospital while awaiting heart transplant or as a destination therapy.

## Conclusion

Albeit randomized evidence supporting its clinical use remains scarce, the Impella™ device is an emerging MCS device for treatment of CS. The Impella™ actively unloads the impaired left or right ventricle and maintains systemic pressure. If immediately applied, these devices not only unload the ventricle but also improve myocardial and peripheral oxygen supply and therefore have the potential to halt the shock spiral and reverse MODS. Owing to its design, the Impella™ relieves the battered ventricle, which appears to improve myocardial recovery. Profound understanding of the device, its physiologic impact, but also its limitations are important when considering a CS patient for percutaneous circulatory support.

## Author Contributions

AA-T has drafted and corrected the manuscript. MB, GC, GT, MM, AB, RK, and FC revised the manuscript critically for important intellectual content. All authors contributed to the article and approved the submitted version.

## Conflict of Interest

MB has received speaker fees from Abbott Vascular, Abiomed, and SIS Medical. FC has received speaker and/or consultant fees from Abbott Vascular, Abiomed, Boston Scientific, and SIS Medical. RK has received institutional grants from Abbott, Biosense-Webster, Biotronik, Boston, Medtronic, SIS Medical, and consultant fees from Biosense-Webster and Biotronik. The remaining authors declare that the research was conducted in the absence of any commercial or financial relationships that could be construed as a potential conflict of interest.

## Publisher's Note

All claims expressed in this article are solely those of the authors and do not necessarily represent those of their affiliated organizations, or those of the publisher, the editors and the reviewers. Any product that may be evaluated in this article, or claim that may be made by its manufacturer, is not guaranteed or endorsed by the publisher.

## Abbreviations

AMI: Acute myocardial infarction
HF: Heart failure
CS: Cardiogenic shock
ECMO: Extracorporeal membrane oxygenation
LVEF: Left ventricular ejection fraction
LVAD: Left ventricular assist device
MCS: Mechanical circulatory support
MODS: Multiorgan dysfunction syndrome
PCI: Percutaneous coronary intervention
pVADs: Percutaneous ventricular assist devices
RHF: Right heart failure
SIRS: Systemic inflammatory response syndrome
STEMI: ST-elevation myocardial infarction.

## References

[1] Mebazaa A Combes A van Diepen S Hollinger A Katz JN Landoni G. Management of cardiogenic shock complicating myocardial infarction. Intensive Care Med. (2018) 44:760–73. 10.1007/s00134-018-5214-9 29767322

[2] Ibanez B James S Agewall S Antunes MJ Bucciarelli-Ducci C Bueno H. 2017 ESC Guidelines for the management of acute myocardial infarction in patients presenting with ST-segment elevation: the task force for the management of acute myocardial infarction in patients presenting with ST-segment elevation of the European society of cardiology (ESC). Eur Heart J. (2018) 39:119–77. 10.1093/eurheartj/ehx393 28886621

[3] van Diepen S Katz JN Albert NM Henry TD Jacobs AK Kapur NK. Contemporary management of cardiogenic shock: a scientific statement from the american heart association. Circulation. (2017) 136:e232–e68. 10.1161/CIR.0000000000000525 28923988

[4] Werdan K Gielen S Ebelt H Hochman JS. Mechanical circulatory support in cardiogenic shock. Eur Heart J. (2014) 35:156–67. 10.1093/eurheartj/eht248 24014384

[5] Touchan J Guglin M. Temporary mechanical circulatory support for cardiogenic shock. Curr Treat Options Cardiovasc Med. (2017) 19:77. 10.1007/s11936-017-0576-9 28913740

[6] McDonagh TA Metra M Adamo M Gardner RS Baumbach A Böhm M. 2021 ESC guidelines for the diagnosis and treatment of acute and chronic heart failure. Eur Heart J. (2021) 42:3599–726. 10.1093/eurheartj/ehab368 34447992

[7] Amin AP Spertus JA Curtis JP Desai N Masoudi FA Bach RG. The evolving landscape of impella use in the united states among patients undergoing percutaneous coronary intervention with mechanical circulatory support. Circulation. (2020) 141:273–84. 10.1161/CIRCULATIONAHA.119.044007 31735078

[8] Hochman JS. Cardiogenic shock complicating acute myocardial infarction: expanding the paradigm. Circulation. (2003) 107:2998–3002. 10.1161/01.CIR.0000075927.67673.F2 12821585

[9] Prondzinsky R Unverzagt S Lemm H Wegener NA Schlitt A Heinroth KM. Interleukin-6,−7,−8, and−10 predict outcome in acute myocardial infarction complicated by cardiogenic shock. Clin Res Cardiol. (2012) 101:375–84. 10.1007/s00392-011-0403-3 22212516

[10] Maack C Eschenhagen T Hamdani N Heinzel FR Lyon AR Manstein DJ. Treatments targeting inotropy. Eur Heart J. (2019) 40:3626–44. 10.1093/eurheartj/ehy600 30295807 PMC7963133

[11] Mebazaa A Motiejunaite J Gayat E Crespo-Leiro MG Lund LH Maggioni AP. Long-term safety of intravenous cardiovascular agents in acute heart failure: results from the European society of cardiology heart failure long-term registry. Eur J Heart Fail. (2018) 20:332–41. 10.1002/ejhf.991 28990358

[12] Vahdatpour C Collins D Goldberg S. Cardiogenic shock. J Am Heart Assoc. (2019) 8:e011991. 10.1161/JAHA.119.011991 30947630 PMC6507212

[13] Thiele H Ohman EM de Waha-Thiele S Zeymer U Desch S. Management of cardiogenic shock complicating myocardial infarction: an update 2019. Eur Heart J. (2019) 40:2671–83. 10.1093/eurheartj/ehz363 31274157

[14] Burzotta F Trani C Doshi SN Townend J van Geuns RJ Hunziker P. Impella ventricular support in clinical practice: collaborative viewpoint from a European expert user group. Int J Cardiol. (2015) 201:684–91. 10.1016/j.ijcard.2015.07.065 26363632

[15] Remmelink M Sjauw KD Henriques JP de Winter RJ Vis MM Koch KT. Effects of mechanical left ventricular unloading by Impella on left ventricular dynamics in high-risk and primary percutaneous coronary intervention patients. Catheter Cardiovasc Interv. (2010) 75:187–94. 10.1002/ccd.22263 19941329

[16] Ward KE Tuggle DW Gessouroun MR Overholt ED Mantor PC. Transseptal decompression of the left heart during ECMO for severe myocarditis. Ann Thorac Surg. (1995) 59:749–51. 10.1016/0003-4975(94)00579-6 7887727

[17] Seib PM Faulkner SC Erickson CC Van Devanter SH Harrell JE Fasules JW. Blade and balloon atrial septostomy for left heart decompression in patients with severe ventricular dysfunction on extracorporeal membrane oxygenation. Catheter Cardiovasc Interv. (1999) 46:179–86. 10.1002/(SICI)1522-726X(199902)46:2&lt;179::AID-CCD13&gt;3.0.CO;2-W 10348539

[18] Thiele H Schuler G Neumann FJ Hausleiter J Olbrich HG Schwarz B. Intraaortic balloon counterpulsation in acute myocardial infarction complicated by cardiogenic shock: design and rationale of the intraaortic balloon pump in cardiogenic shock II (IABP-SHOCK II) trial. Am Heart J. (2015) 169:e7–8. 10.1016/j.ahj.2015.01.009 25819870

[19] Ahmad Y Sen S Shun-Shin MJ Ouyang J Finegold JA Al-Lamee RK. Intra-aortic balloon pump therapy for acute myocardial infarction: a meta-analysis. JAMA Intern Med. (2015) 175:931–9. 10.1001/jamainternmed.2015.0569 25822657

[20] Valgimigli M Steendijk P Sianos G Onderwater E Serruys PW. Left ventricular unloading and concomitant total cardiac output increase by the use of percutaneous Impella Recover LP 2. 5 assist device during high-risk coronary intervention catheter. Cardiovasc Interv. (2005) 65:263–7. 10.1002/ccd.20380 15895406

[21] Suga H Hayashi T Shirahata M. Ventricular systolic pressure-volume area as predictor of cardiac oxygen consumption. Am J Physiol. (1981) 240:H39–44. 10.1152/ajpheart.1981.240.1.H39 7457620

[22] Suga H. Total mechanical energy of a ventricle model and cardiac oxygen consumption. Am J Physiol. (1979) 236:H498–505. 10.1152/ajpheart.1979.236.3.H498 426086

[23] Sarnoff SJ Braunwald E Welch GH Case RB Stainsby WN Macruz R. Hemodynamic determinants of oxygen consumption of the heart with special reference to the tension-time index. Am J Physiol. (1958) 192:148–56. 10.1152/ajplegacy.1957.192.1.148 13498167

[24] Braunwald E. 50th anniversary historical article. Myocardial oxygen consumption: the quest for its determinants and some clinical fallout. J Am Coll Cardiol. (1999) 34:1365–8. 10.1016/S0735-1097(99)00428-3 10551680

[25] Burzotta F Paloscia L Trani C Mascellanti M Mongiardo R Materazzo G. Feasibility and long-term safety of elective Impella-assisted high-risk percutaneous coronary intervention: a pilot two-centre study. J Cardiovasc Med. (2008) 9:1004–10. 10.2459/JCM.0b013e3282f9abe7 18799962

[26] Dixon SR Henriques JP Mauri L Sjauw K Civitello A Kar B. A prospective feasibility trial investigating the use of the Impella 2. 5 system in patients undergoing high-risk percutaneous coronary intervention (The PROTECT I Trial): initial US experience JACC. Cardiovasc Interv. (2009) 2:91–6. 10.1016/j.jcin.2008.11.005 19463408

[27] Remmelink M Sjauw KD Henriques JP de Winter RJ Koch KT van der Schaaf RJ. Effects of left ventricular unloading by Impella recover LP2. 5 on coronary hemodynamics catheter. Cardiovasc Interv. (2007) 70:532–7. 10.1002/ccd.21160 17896398

[28] Sauren LD Accord RE Hamzeh K de Jong M van der Nagel T van der Veen FH. Combined Impella and intra-aortic balloon pump support to improve both ventricular unloading and coronary blood flow for myocardial recovery: an experimental study. Artif Organs. (2007) 31:839–42. 10.1111/j.1525-1594.2007.00477.x 18001394

[29] Reesink KD Dekker AL Van Ommen V Soemers C Geskes GG van der Veen FH. Miniature intracardiac assist device provides more effective cardiac unloading and circulatory support during severe left heart failure than intraaortic balloon pumping. Chest. (2004) 126:896–902. 10.1378/chest.126.3.896 15364772

[30] Burkhoff D Sayer G Doshi D Uriel N. Hemodynamics of mechanical circulatory support. J Am Coll Cardiol. (2015) 66:2663–74. 10.1016/j.jacc.2015.10.017 26670067

[31] Rao P Khalpey Z Smith R Burkhoff D Kociol RD. Venoarterial extracorporeal membrane oxygenation for cardiogenic shock and cardiac arrest. Circ Heart Fail. (2018) 11:e004905. 10.1161/CIRCHEARTFAILURE.118.004905 30354364

[32] Meyns B Dens J Sergeant P Herijgers P Daenen W Flameng W. Initial experiences with the Impella device in patients with cardiogenic shock - Impella support for cardiogenic shock. Thorac Cardiovasc Surg. (2003) 51:312–7. 10.1055/s-2003-45422 14669126

[33] Aqel RA Hage FG Iskandrian AE. Improvement of myocardial perfusion with a percutaneously inserted left ventricular assist device. J Nucl Cardiol. (2010) 17:158–60. 10.1007/s12350-009-9127-4 19685267

[34] Konstam MA Kiernan MS Bernstein D Bozkurt B Jacob M Kapur NK. Evaluation and Management of Right-Sided Heart Failure: A Scientific Statement From the American Heart Association. Circulation. (2018) 137:e578–622. 10.1161/CIR.0000000000000560 29650544

[35] Kapur NK Esposito ML Bader Y Morine KJ Kiernan MS Pham DT. Mechanical circulatory support devices for acute right ventricular failure. Circulation. (2017) 136:314–26. 10.1161/CIRCULATIONAHA.116.025290 28716832

[36] Harjola VP Mebazaa A Celutkiene J Bettex D Bueno H Chioncel O. Contemporary management of acute right ventricular failure: a statement from the heart failure association and the working group on pulmonary circulation and right ventricular function of the European society of cardiology. Eur J Heart Fail. (2016) 18:226–41. 10.1002/ejhf.478 26995592

[37] Anderson MB Goldstein J Milano C Morris LD Kormos RL Bhama J. Benefits of a novel percutaneous ventricular assist device for right heart failure: the prospective RECOVER RIGHT study of the Impella RP device. J Heart Lung Transplant. (2015) 34:1549–60. 10.1016/j.healun.2015.08.018 26681124

[38] Walker LA Buttrick PM. The right ventricle: biologic insights and response to disease. Curr Cardiol Rev. (2009) 5:22–8. 10.2174/157340309787048077 20066144 PMC2803284

[39] Lampert BC Teuteberg JJ. Right ventricular failure after left ventricular assist devices. J Heart Lung Transplant. (2015) 34:1123–30. 10.1016/j.healun.2015.06.015 26267741

[40] Anderson M Morris DL Tang D Batsides G Kirtane A Hanson I. Outcomes of patients with right ventricular failure requiring short-term hemodynamic support with the Impella RP device. J Heart Lung Transplant. (2018) 37:1448–58. 10.1016/j.healun.2018.08.001 30241890

[41] Abiomed Press Releases. Impella RP Post-Approval Study Data Presented at ACC 2019. Available online at: http://investors.abiomed.com/news-releases/news-release-details/impella-rp-post-approval-study-data-presented-acc-2019. (accessed March 19, 2019).

[42] Haneya A Philipp A Puehler T Rupprecht L Kobuch R Hilker M. Temporary percutaneous right ventricular support using a centrifugal pump in patients with postoperative acute refractory right ventricular failure after left ventricular assist device implantation. Eur J Cardiothorac Surg. (2012) 41:219–23. 10.1016/j.ejcts.2011.04.029 21641814 PMC3241097

[43] Yan I Grahn H Blankenberg S Westermann D. Right ventricular temporal assist device for cardiac recompensation. ESC Heart Fail. (2017) 4:376–8. 10.1002/ehf2.12148 28772056 PMC5542743

[44] Morgan JA O'Neill WW. Percutaneous right ventricular assist device support in a patient supported by an LVAD. ASAIO J. (2016) 62:e41–2. 10.1097/MAT.0000000000000344 26771398

[45] Kuchibhotla S Esposito ML Breton C Pedicini R Mullin A O'Kelly R. Acute biventricular mechanical circulatory support for cardiogenic shock. J Am Heart Assoc. (2017) 6:6670. 10.1161/JAHA.117.006670 29054842 PMC5721869

[46] Seyfarth M Sibbing D Bauer I Fröhlich G Bott-Flügel L Byrne R. A randomized clinical trial to evaluate the safety and efficacy of a percutaneous left ventricular assist device vs. intra-aortic balloon pumping for treatment of cardiogenic shock caused by myocardial infarction. J Am Coll Cardiol. (2008) 52:1584–8. 10.1016/j.jacc.2008.05.065 19007597

[47] Ouweneel DM Eriksen E Sjauw KD van Dongen IM Hirsch A Packer EJ. Percutaneous mechanical circulatory support vs. intra-aortic balloon pump in cardiogenic shock after acute myocardial infarction. J Am Coll Cardiol. (2017) 69:278–87. 10.1016/j.jacc.2016.10.022 27810347

[48] Basir MB Kapur NK Patel K Salam MA Schreiber T Kaki A. Improved outcomes associated with the use of shock protocols: updates from the national cardiogenic shock initiative. Catheter Cardiovasc Interv. (2019) 93:1173–83. 10.1002/ccd.28307 31025538

[49] Tehrani BN Truesdell AG Sherwood MW Desai S Tran HA Epps KC. Standardized team-based care for cardiogenic shock. J Am Coll Cardiol. (2019) 73:1659–69. 10.1016/j.jacc.2018.12.084 30947919

[50] Schrage B Ibrahim K Loehn T Werner N Sinning JM Pappalardo F. Impella support for acute myocardial infarction complicated by cardiogenic shock. Circulation. (2019) 139:1249–58. 10.1161/CIRCULATIONAHA.118.036614 30586755

[51] Dhruva SS Ross JS Mortazavi BJ Hurley NC Krumholz HM Curtis JP. Association of use of an intravascular microaxial left ventricular assist device vs. Intra-aortic balloon pump with in-hospital mortality and major bleeding among patients with acute myocardial infarction complicated by cardiogenic shock. JAMA. (2020) 323:734–45. 10.1001/jama.2020.0254 32040163 PMC7042879

[52] Iannaccone M Albani S Giannini F Colangelo S Boccuzzi GG Garbo R. Short term outcomes of Impella in cardiogenic shock: a review and meta-analysis of observational studies. Int J Cardiol. (2021) 324:44–51. 10.1016/j.ijcard.2020.09.044 32971148

[53] Karami M den Uil CA Ouweneel DM Scholte NT Engström AE Akin S. Mechanical circulatory support in cardiogenic shock from acute myocardial infarction: Impella CP/5. *0 vs.* ECMO. Eur Heart J Acute Cardiovasc Care. (2020) 9:164–72. 10.1177/2048872619865891 31353918

[54] Badawi RA Grise MA Thornton SN. Impella 2. 5 assisted balloon aortic valvuloplasty and percutaneous coronary intervention as a bridge to heart transplantation. J Invasive Cardiol. (2012) 24:229–30. 10.1016/j.jvs.2021.09.036 22562918

[55] Megaly M Jones P. Impella CP-assisted balloon aortic valvuloplasty. J Cardiol Cases. (2016) 14:49–51. 10.1016/j.jccase.2016.03.012 30546662 PMC6282924

[56] Karatolios K Chatzis G Luesebrink U Markus B Ahrens H Tousoulis D. Impella support following emergency percutaneous balloon aortic valvuloplasty in patients with severe aortic valve stenosis and cardiogenic shock. Hellenic J Cardiol. (2019) 60:178–81. 10.1016/j.hjc.2018.02.008 29571667

[57] Spiro J Venugopal V Raja Y Ludman PF Townend JN Doshi SN. Feasibility and efficacy of the 2. 5 L and 38 L impella percutaneous left ventricular support device during high-risk, percutaneous coronary intervention in patients with severe aortic stenosis catheter. Cardiovasc Interv. (2015) 85:981–9. 10.1002/ccd.25355 24408882

[58] Martinez CA Singh V Londoño JC Cohen MG Alfonso CE O'Neill WW. Percutaneous retrograde left ventricular assist support for interventions in patients with aortic stenosis and left ventricular dysfunction. Catheter Cardiovasc Interv. (2012) 80:1201–9. 10.1002/ccd.24303 22511541

[59] Singh V Damluji AA Mendirichaga R Alfonso CE Martinez CA Williams D. Elective or emergency use of mechanical circulatory support devices during transcatheter aortic valve replacement. J Interv Cardiol. (2016) 29:513–22. 10.1111/joic.12323 27550213

[60] Burzotta F Nerla R Trani C. Bail-out use of Impella cp as a bridge to TAVI in a cardiogenic shock patient: the “pump-rewiring” technique. J Invasive Cardiol. (2016) 28:E1–5. 26716594

[61] O'Neill WW Grines C Schreiber T Moses J Maini B Dixon SR. Analysis of outcomes for 15,259 US patients with acute myocardial infarction cardiogenic shock (AMICS) supported with the Impella device. Am Heart J. (2018) 202:33–8. 10.1016/j.ahj.2018.03.024 29803984

[62] Acharya D Gulack BC Loyaga-Rendon RY Davies JE He X Brennan JM. clinical characteristics and outcomes of patients with myocardial infarction and cardiogenic shock undergoing coronary artery bypass surgery: data from the society of thoracic surgeons national database. Ann Thorac Surg. (2016) 101:558–66. 10.1016/j.athoracsur.2015.10.051 26718859 PMC5142520

[63] Pepino P Coronella G Oliviero P Monaco M Schiavone V Finizio F. Successful use of the Impella recover LP 5.0 device for circulatory support during off-pump coronary artery bypass grafting. Int J Surg Case Rep. (2014) 5:803–5. 10.1016/j.ijscr.2014.07.013 25305600 PMC4245662

[64] Akay MH Frazier OH. Impella recover 5. 0 assisted coronary artery bypass grafting. J Card Surg. (2010) 25:606–7. 10.1111/j.1540-8191.2010.01071.x 20662974

[65] Gregoric ID Poglajen G Span M Frazier OH Loyalka P Kar B. Percutaneous ventricular assist device support during off-pump surgical coronary revascularization. Ann Thorac Surg. (2008) 86:637–9. 10.1016/j.athoracsur.2008.01.038 18640347

[66] Pae WE Miller CA Matthews Y Pierce WS. Ventricular assist devices for postcardiotomy cardiogenic shock. A combined registry experience. J Thorac Cardiovasc Surg. (1992) 104:541–52. discussion 52–3. 10.1016/S0022-5223(19)34717-8 1513144

[67] Samuels LE Kaufman MS Thomas MP Holmes EC Brockman SK Wechsler AS. Pharmacological criteria for ventricular assist device insertion following postcardiotomy shock: experience with the Abiomed BVS system. J Card Surg. (1999) 14:288–93. 10.1111/j.1540-8191.1999.tb00996.x 10874615

[68] Siegenthaler MP Brehm K Strecker T Hanke T Nötzold A Olschewski M. The Impella Recover microaxial left ventricular assist device reduces mortality for postcardiotomy failure: a three-center experience. J Thorac Cardiovasc Surg. (2004) 127:812–22. 10.1016/j.jtcvs.2003.09.055 15001911

[69] Thomas MP Altman A Magovern GJ Moraca RJ. Insertion of an Abiomed Impella® left ventricular assist device following bioprosthetic aortic valve placement. J Card Surg. (2013) 28:469–71. 10.1111/jocs.12118 23675749

[70] Subramaniam AV Barsness GW Vallabhajosyula S. Complications of temporary percutaneous mechanical circulatory support for cardiogenic shock: an appraisal of contemporary literature. Cardiol Ther. (2019) 8:211–28. 10.1007/s40119-019-00152-8 31646440 PMC6828896

[71] Ouweneel DM de Brabander J Karami M Sjauw KD Engstrom AE Vis MM. Real-life use of left ventricular circulatory support with Impella in cardiogenic shock after acute myocardial infarction: 12 years AMC experience. Eur Heart J Acute Cardiovasc Care. (2019) 8:338–49. 10.1177/2048872618805486 30403366 PMC6616211

[72] Boll G Fischer A Kapur NK Salehi P. Right Axillary Artery Conduit Is a Safe and Reliable Access for Implantation of Impella 5. 0 Microaxial Pump. Ann Vasc Surg. (2019) 54:54–9. 10.1016/j.avsg.2018.10.004 30339902

[73] Pappalardo F Scandroglio AM Latib A. Full percutaneous biventricular support with two Impella pumps: the Bi-Pella approach. ESC Heart Fail. (2018) 5:368–71. 10.1002/ehf2.12274 29465166 PMC5933967

[74] Aghili N Bader Y Vest AR Kiernan MS Kimmelstiel C DeNofrio D. Biventricular circulatory support using 2 axial flow catheters for cardiogenic shock without the need for surgical vascular access. Circ Cardiovasc Interv. (2016) 9:3636. 10.1161/CIRCINTERVENTIONS.116.003636 27188188

[75] Chiu CY Hättasch R Praeger D Knebel F Stangl K Ramirez ID. Percutaneous biventricular Impella support in therapy-refractory cardiogenic shock. Heart Lung. (2018) 47:250–2. 10.1016/j.hrtlng.2018.03.009 29628145

[76] Hunziker P Hunziker L. Percutaneous biventricular cardiac assist device in cardiogenic shock. Eur Heart J. (2013) 34:1620. 10.1093/eurheartj/eht020 23594594

[77] Kamioka N Patel A Burke MA Greenbaum A Babaliaros V. Biventricular Impella placement via complete venous access. Catheter Cardiovasc Interv. (2019) 93:E343–E5. 10.1002/ccd.27103 28544381

[78] Kapur NK Jumean M Ghuloom A Aghili N Vassallo C Kiernan MS. First successful use of two axial flow catheters for percutaneous biventricular circulatory support as a bridge to a durable left ventricular assist device. Circ Heart Fail. (2015) 8:1006–8. 10.1161/CIRCHEARTFAILURE.115.002374 26374919

[79] Pyles LA Gustafson RA Fortney J Einzig S. Extracorporeal membrane oxygenation induced cardiac dysfunction in newborn lambs. J Cardiovasc Transl Res. (2010) 3:625–34. 10.1007/s12265-010-9215-5 20848344

[80] Ostadal P Mlcek M Kruger A Hala P Lacko S Mates M. Increasing venoarterial extracorporeal membrane oxygenation flow negatively affects left ventricular performance in a porcine model of cardiogenic shock. J Transl Med. (2015) 13:266. 10.1186/s12967-015-0634-6 26275717 PMC4537539

[81] Ise H Kitahara H Aubin H Saeed D Westenfeld R Akhyari P. Additional unloading of the left ventricle using the Impella LP 2.5 during extracorporeal life support in cases of pulmonary congestion. J Surg Case Rep. (2018) 2018:rjy302. 10.1093/jscr/rjy302 30443318 PMC6232276

[82] Jouan J Grinda JM Bricourt MO Cholley B Fabiani JN. Successful left ventricular decompression following peripheral extracorporeal membrane oxygenation by percutaneous placement of a micro-axial flow pump. J Heart Lung Transplant. (2010) 29:135–6. 10.1016/j.healun.2009.06.007 19782598

[83] Cheng A Swartz MF Massey HT. Impella to unload the left ventricle during peripheral extracorporeal membrane oxygenation. ASAIO J. (2013) 59:533–6. 10.1097/MAT.0b013e31829f0e52 23995997

[84] Pappalardo F Schulte C Pieri M Schrage B Contri R Soeffker G. Concomitant implantation of Impella. Eur J Heart Fail. (2017) 19:404–12. 10.1002/ejhf.668 27709750

[85] Cubeddu RJ Lago R Horvath SA Vignola PA O'Neill W Palacios IF. Use of the Impella 2. 5 system alone, after and in combination with an intra-aortic balloon pump in patients with cardiogenic shock: case description and review of the literature. EuroIntervention. (2012) 7:1453–60. 10.4244/EIJV7I12A226 22522555

[86] Udesen NJ Moller JE Lindholm MG Eiskjaer H Schafer A Werner N. Rationale and design of DanGer shock: Danish-German cardiogenic shock trial. Am Heart J. (2019) 214:60–8. 10.1016/j.ahj.2019.04.019 31176289

[87] Esposito ML Zhang Y Qiao X Reyelt L Paruchuri V Schnitzler GR. Left ventricular unloading before reperfusion promotes functional recovery after acute myocardial infarction. J Am Coll Cardiol. (2018) 72:501–14. 10.1016/j.jacc.2018.05.034 30049311 PMC6817809

[88] Kapur NK Qiao X Paruchuri V Morine KJ Syed W Dow S. Mechanical pre-conditioning with acute circulatory support before reperfusion limits infarct size in acute myocardial infarction. JACC Heart Fail. (2015) 3:873–82. 10.1016/j.jchf.2015.06.010 26541785

